# Impact of metabolic score for visceral fat on bone health in adolescent population: a cross-sectional study perspective

**DOI:** 10.3389/fnut.2026.1822235

**Published:** 2026-06-30

**Authors:** Linlin Chen, Jianxi Liu, Longkai Shi, Yingli Xu, Wenqing Ding

**Affiliations:** 1 School of Public Health, Ningxia Medical University, Yinchuan, China; 2 Key Laboratory of Environmental Factors and Chronic Disease Control, Yinchuan, China

**Keywords:** adolescents, bone metabolism markers, bone mineral content, osteoporosis, the metabolic score for visceral fat

## Abstract

**Objective:**

To analyze the relationship between the Metabolic Score for Visceral Fat (METS-VF), bone mineral content (BMC), and bone metabolism indexes in Chinese adolescents.

**Methods:**

This cross-sectional study used stratified cluster sampling to select 1,206 adolescents (12–18 years) from six schools in Yinchuan (2017–2023). Body composition and BMC of the participants were measured using bioelectrical impedance analysis (BIA). Serum bone metabolism indexes [osteocalcin (OC) and C-terminal telopeptide of type I collagen (CTX)] were measured.

**Results:**

METS-VF was found to be associated with BMC (*r* = 0.484, *P* < 0.001) and OC (*r* = −0.074, *P* = 0.010), but not with CTX (*r* = 0.042, *P* = 0.141). After adjusting for confounding factors, compared with the lowest quartile of METS-VF, the risk of low BMC in the highest quartile was significantly lower, with an OR of 0.17 (95% CI: 0.05–0.49), whereas the risk of low OC was significantly higher, with an OR of 3.47 (95% CI: 1.82–6.59) (all *P* < 0.05). In sex-stratified analyses, this association was significant in both boys and girls (OR_boys_ = 0.03, 95% CI: 0.01–0.46, *P* = 0.007; OR_girls_ = 0.20, 95% CI: 0.06–0.64, *P* = 0.005). Restricted cubic spline curves indicated that there was an inverted U-shaped non-linear relationship between METS-VF and BMC (*P-*overall = 0.002, *P*-nonlinear = 0.009). In sex-stratified analyses, the association was significant in girls (*P*-nonlinear = 0.152; *P-*overall = 0.008); Moreover, linear relationship between METS-VF and OC were found (*P* nonlinear = 0.162, *P-*overall <0.05). Receiver operating characteristic (ROC) curves showed that METS-VF had modest discriminatory ability for low BMC better than VAI and TyG (AUC = 0.641, 95% CI = 0.605–0.678, *P* < 0.05).

**Conclusion:**

Our findings suggest a non-linear association between METS-VF and BMC in adolescents, and fat distribution and content were more closely related to bone metabolism.

## 1 Introduction

Osteoporosis (OP) is a disease characterized by reduced bone mass and an increased risk of fractures, representing a significant public health burden globally (1). Adolescence is a critical period for bone mass accrual, with over 90% of peak bone mass attained by the end of this phase (2, 3). Therefore, suboptimal bone accumulation during adolescence predisposes individuals to lifelong skeletal fragility, highlighting the importance of identifying modifiable determinants of bone health in this population.

While obesity has traditionally been viewed as protective against osteoporosis due to mechanical loading effects, emerging evidence suggests a paradoxical relationship between adipose tissue distribution and bone metabolism (4–6). Body mass index (BMI), a commonly used measure of obesity, fails to differentiate between visceral and subcutaneous fat nor does it account for variations in lean mass. This limitation obscures the true biological interplay between adiposity and bone (7, 8). Visceral adipose tissue (VAT), which is metabolically active and a potent source of pro-inflammatory cytokines (IL-1,6, TNF-*α*) and adipokines (leptin, adiponectin), has been implicated in bone remodeling through both direct and indirect pathways (9, 10). Notably, factors derived from VAT may suppress osteoblast activity while promoting osteoclastogenesis, potentially offsetting the mechanical benefits of overall adiposity (11). The Metabolic Score for Visceral Fat (METS-VF), a novel composite index of visceral adiposity that integrates the metabolic score for insulin resistance (METS-IR), waist-to-height ratio (WHtR), age, and sex, has demonstrated superior predictive validity for visceral adiposity compared to traditional measures (12). This indicator has been validated in various diseases such as cardiovascular disease, diabetes, and hypertension (13–15). Bone growth and turnover are markedly increased during adolescence. This developmental period coincides with hormonal changes that may amplify the metabolic effects of visceral adiposity on bone metabolism. Serum biomarkers such as osteocalcin (OC), a marker of bone formation, and C-terminal telopeptide of type I collagen (CTX), a marker of bone resorption, provide dynamic insights into bone turnover. However, their associations with METS-VF remain uncharacterized in adolescents.

Therefore, this study aimed to investigate the relationship between METS-VF and BMC, OC, and CTX to elucidate the impact of visceral adiposity on adolescent bone health.

## 2 Methods

### 2.1 Study population

**Figure 1:**
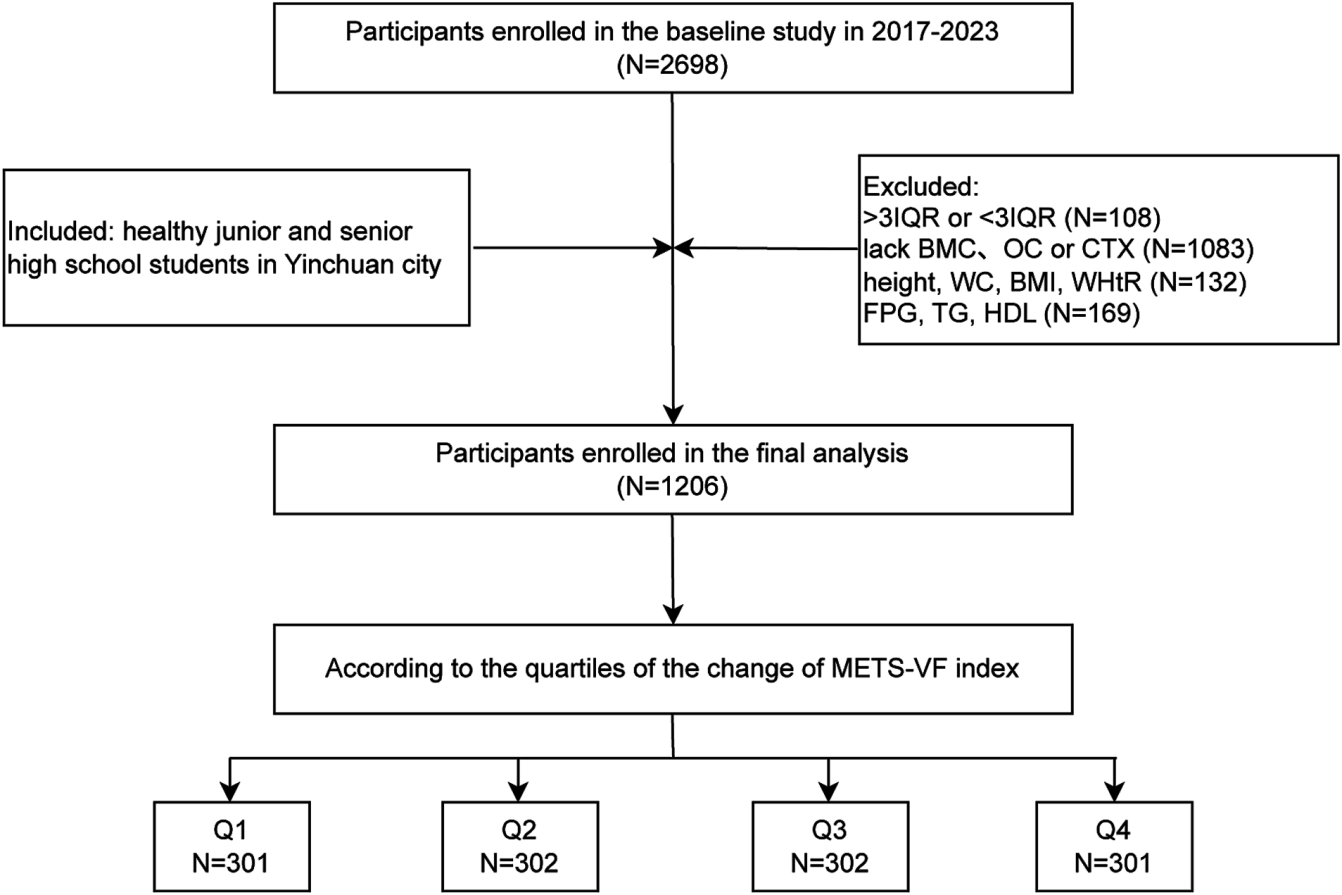
Flowchart of the study population. FPG, Fasting plasma glucose; TG, Triglyceride; HDL-C, High-density lipoprotein cholesterol; BMI, body mass index; BMC, bone mineral content; OC, osteocalcin; CTX, C-terminal telopeptide of type I collagen.

This serial cross-sectional study employed a stratified cluster sampling to ensure representative inclusion of adolescents from Yinchuan, China, between 2017 and 2023. First, six schools (three junior and three senior high schools) were selected and then stratified by grade. Finally, classes were randomly selected from each grade: 13 classes from junior high schools and 32 classes from senior high schools. All of them finished the questionnaires, anthropometric measurements, blood sample collection, and clinical examinations. Exclusion criteria:(1) Age outside the range of 12 to 18 years; (2) Missing data on waist circumference (WC), fasting plasma glucose (FPG), triglycerides (TG), BMI, high-density lipoprotein cholesterol (HDL-C), BMC, OC and CTX; Specific results are shown in the flowchart in Figure 1.

The study was approved by the Ningxia Medical University Ethics Committee (2016–123 and 2021-G053) and conducted by the Declaration of Helsinki. Written informed consent was secured from all participants as well as their legal guardians.

### 2.2 Questionnaire investigation

A standard self-administered questionnaire was used to collect the following information: basic demographics (name, age, sex, parental education, and family status), lifestyle (eating breakfast, drinking, smoking status, and sleep), and physical activity. Smoking was defined as ≥1 cigarette in the past 30 days while alcohol consumption was defined as ≥1 alcoholic drink in the past 30 days. Sleep time was calculated from self-reported bedtime and wake time on school days. Physical activity was assessed via the International Physical Activity Questionnaire-Short Form (IPAQ-SF), modified for adolescents (16). Participants reported the frequency and duration of moderate-to-vigorous physical activities (MVPA) causing sweating or increased heart rate (≥30 min/session).

### 2.3 Anthropometric data

All anthropometric variables were measured twice by the same trained physician after the adolescents had taken off their shoes and heavy clothing. Height and WC were measured with an accuracy of 0.1 cm; and the error between the two measurements was less than 0.5 cm. Weight was measured with an accuracy of 0.1 kg, and the error between the two measurements was less than 0.5 kg. The mean values of weight and height were used to calculate BMI, which is calculated as weight (kg)/ height^2^ (m^2^). The WHtR was derived as WC (cm)/height (cm).

### 2.4 Bone mineral content (BMC) determination and METS-VF definition

BMC was obtained using an InBody-370 body composition analyzer (Biospace, Korea) based on bioelectrical impedance analysis (BIA). The BMC value was the BIA-derived estimated bone mineral content reported by the InBody-370 body composition analyzer. BIA is a simple, non-invasive, radiation-free, and relatively affordable method that is suitable for large-scale epidemiological studies of body composition (17). Low BMC was defined as BMC Z-score≤-1SD (18). Low OC and low CTX were defined as values below the 25th percentile of their respective distributions, whereas values at or above the 25th percentile were classified as high OC and high CTX.

METS-VF = 4.466 + 0.011{[Ln (METS-IR)]^3^} + 3.239{[Ln (WHtR)]^3^} + 0.319(sex) + 0.594 [Ln (Age)] (12). (male = 1, female = 0); Age was expressed in years.

METS-IR = Ln [2*FPG (mg/dL) + TG (mg/dL)] *BMI/Ln HDL-C(mg/dL) (12).

### 2.5 Biochemical measurements

Fasting venous blood (3 mL) was collected from participants after an 8-h overnight fast and centrifuged at 3,000 rpm for 10 min (TDL-5-A, centrifugal radius 17.9 cm). OC and type I collagen cross-linked CTX were quantified using human OC and CTX enzyme-linked immunosorbent assay (ELISA) kits, respectively. The results were recorded on a full-wavelength multifunction microplate reader (Multiskan GO type), and the intra-plate and inter-plate coefficients of variation (CVs) were rigorously controlled below 10%.

### 2.6 Statistical analysis

Data were analyzed using SPSS 26.0, R 4.2.2, and GraphPad Prism 8.0.2. Continuous variables are reported as mean ± standard deviation (SD) or median with interquartile range (IQR, 25th–75th percentiles), while Categorical variables are expressed as frequencies and percentages (%). Baseline characteristics were compared using ANOVA tests (for multi-group comparisons), Kruskal-Wallis tests (for non-parametric data), and chi-square tests (for categorical variables), as appropriate. Firth penalized logistic regression models assessed associations between METS-VF (quartiles) and bone parameters (BMC, OC, CTX), adjusted for age, sex, physical activity, smoking, and alcohol consumption. Restricted cubic splines (RCS) evaluated non-linear relationships, and receiver operating characteristic (ROC) curves compared the predictive performance of obesity-related indices. A two-sided *P*-value<0.05 was considered statistically significant.

## 3 Results

### 3.1 General characteristics of study participants

General characteristics stratified by the METS-VF quartiles (quartile1: <4.20; quartile2: 4.20–4.69; quartile3: 4.70–5.33; quartile4: >5.33) are shown in Table 1. The study included 1,206 adolescents with an average age of 14.70 ± 1.26 years, including 680 boys (56.4%). Table 1 shows that there were statistically significant differences in height, weight, BMI, WC, SBP, DBP, FPG, TC, TG, HDL-C, low-density lipoprotein cholesterol (LDL-C), BMC and physical activity among METS-VF quartiles. (all *P*-values<0.05). A comparison of General characteristics between participants included in and excluded from the final analytic sample is shown in Supplementary Table S1.

**Table 1:** General characteristics of study participants stratified by METS-VF.

Characteristics	Overall	Quartiles of METS-VF				*P*-value
		Quartile 1 (<4.20)	Quartile 2 (4.20–4.69)	Quartile 3 (4.70–5.33)	Quartile 4 (>5.33)	
*N* (%)	1,206	301 (25%)	302 (25%)	302 (25%)	301 (25%)	
Age (years)	14.70 ± 1.27	14.41 ± 1.29	14.64 ± 1.29	14.87 ± 1.27	14.89 ± 1.15	**<0.001**
Sex, *n* (%)						<0.001
Male	680 (56.4)	211 (70.1)	156 (51.7)	138 (45.7)	175 (58.1)	
Female	526 (43.6)	90 (29.2)	146 (48.3)	164 (54.3)	126 (41.9)	
Height (cm)	168.34 ± 8.58	170.36 ± 8.60	166.76 ± 8.32	166.58 ± 8.59	169.68 ± 8.20	**<0.001**
Weight (Kg)	58.65 ± 13.44	49.61 ± 6.95	52.11 ± 7.01	57.70 ± 7.67	75.22 ± 12.97	**<0.001**
BMI(Kg/m^2^)	20.60 ± 3.94	17.01 ± 1.22	18.66 ± 1.19	20.71 ± 1.39	26.02 ± 3.35	**<0.001**
WC (cm)	75.15 ± 10.66	65.85 ± 2.87	69.66 ± 3.03	75.31 ± 3.95	89.78 ± 9.57	**<0.001**
SBP (mmHg)	113.09 ± 11.74	110.40 ± 11.84	109.49 ± 10.01	112.03 ± 11.51	119.88 ± 11.43	**<0.001**
DBP (mmHg)	70.02 ± 7.90	67.94 ± 7.54	68.84 ± 7.58	70.15 ± 7.68	72.96 ± 8.00	**<0.001**
FPG (mg/dL)	90.45 ± 10.69	89.48 ± 10.40	90.40 ± 9.80	90.29 ± 11.34	91.94 ± 11.09	**<0.001**
TC (mg/dL)	146.45 ± 28.43	134.35 ± 20.56	141.29 ± 23.89	148.79 ± 28.44	161.38 ± 32.20	**<0.001**
TG^a^ (mg/dL)	79.71 (62.88,105.40)	69.97 (58.01,89.90)	75.73 (60.89,94.77)	80.16 (61.11,107.17)	99.20 (73.07,131.08)	**<0.001**
HDL-C(mg/dL)	52.58 ± 10.21	52.50 ± 9.71	53.31 ± 10.26	53.24 ± 10.96	51.25 ± 9.79	**<0.001**
LDL-C (mg/dL)	73.24 ± 24.03	63.60 ± 16.86	69.31 ± 19.79	75.02 ± 24.23	85.06 ± 28.30	**<0.001**
BMC (Kg)	2.63 ± 0.51	2.46 ± 0.38	2.45 ± 0.40	2.58 ± 0.44	3.05 ± 0.54	**<0.001**
OC (ng/mL)	20.14 ± 13.87	21.52 ± 14.51	20.21 ± 13.39	20.31 ± 13.12	18.51 ± 14.30	0.066
CTX (pg/mL)	2344.93 ± 1751.31	2309.20 ± 1735.07	2251.09 ± 1772.29	2275.87 ± 1663.58	2544.08 ± 1823.73	0.148
Physical activity^b^ *n* (%)						0.042
LPA	152 (36.9)	46 (38.3)	44 (42.7)	40 (38.1)	22 (26.2)	
MPA	132 (31.8)	34 (28.3)	24 (23.3)	34 (32.4)	39 (46.4)	
VPA	129 (31.3)	40 (33.3)	35 (34.0)	31 (29.5)	23 (27.4)	
Smoking^b^ *n* (%)						0.189
Yes	206 (17.9)	63 (22.1)	47 (16.4)	47 (15.9)	49 (17.1)	
No	947 (82.1)	222 (77.9)	240 (83.6)	248 (84.1)	237 (82.9)	
Alcohol consumption^b^ *n* (%)						0.364
Yes	367 (31.9)	101 (35.2)	83 (28.9)	89 (30.4)	94 (33.3)	
No	782 (68.1)	186 (64.8)	204 (71.1)	204 (69.6)	188 (66.7)	

BMI, body mass index; WC, waist circumference; SBP, Systolic blood pressure; DBP, Diastolic blood pressure; FPG, Fasting plasma glucose; TC, Total cholesterol; TG^a^, Triglyceride; HDL-C, High-density lipoprotein cholesterol; LDL-C, Low-density lipoprotein cholesterol; bone mineral content; OC, osteocalcin; CTX, C-terminal telopeptide of type I collagen; LPA, Light-intensity Activity. MPA, Moderate-intensity Activity; VPA, Vigorous-intensity activity.

Data are presented as means ± SD or median (interquartile range) for continuous variables and numbers (%) for categorical variables.

*P*-values are calculated by ANOVA and Kruskal–Wallis tests for continuous variables and Chi-square tests for categorical variables.

ᵃThe *P*-value for TG was calculated using the Kruskal–Wallis test.

ᵇThe number of participants with available data differed across variables because of missing questionnaire data: smoking, *n* = 1,153; alcohol consumption, *n* = 1,149; physical activity, *n* = 413.

Bold values indicate statistical significance (P < 0.05).

### 3.2 Associations of METS-VF quartiles with bone parameters in adolescents

**Figure 2:**
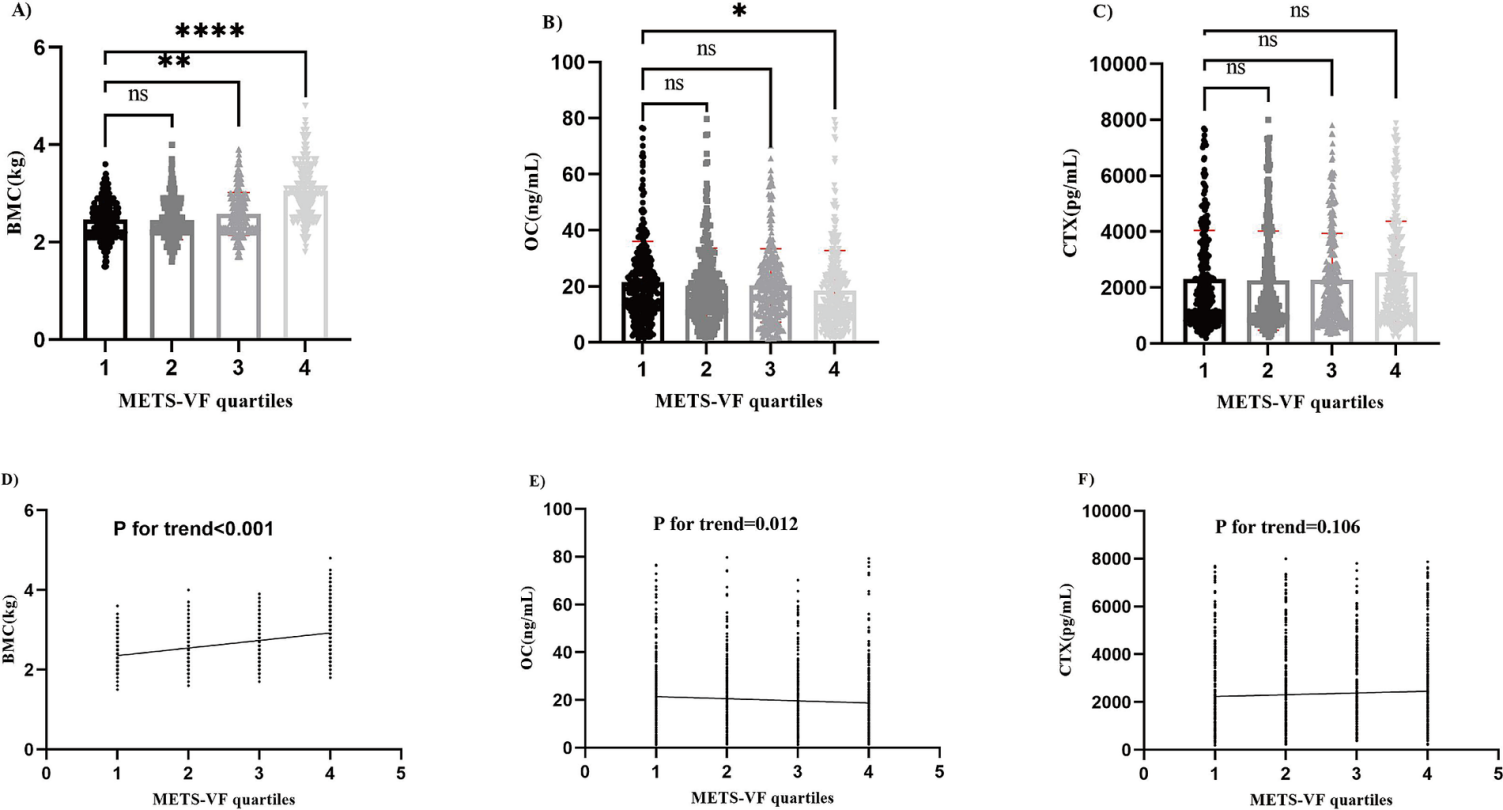
Quartile comparison and trend analyses of increasing METS-VF quartiles for BMC **(A, D)**, OC **(B, E)**, and CTX **(C, F)** in adolescents. METS-VF, metabolic score for Visceral fat; BMC, bone mineral content; OC, osteocalcin; CTX, C-terminal telopeptide of type I collagen. ^ns^*P*-vaule>0.05, *P-value<0.05, **P-value<0.01,****P-value<0.0001.

To explore the relationship between visceral fat and bone parameters, we explored the correlations between METS-VF and BMC, OC, and CTX. METS-VF was found to be associated with BMC (*r* = 0.484, *P <* 0.001) and OC (*r* = −0.074, *P* = 0.010), but not with CTX (*r* = 0.042, *P* = 0.141). Across METS-VF quartiles, we observed that BMC values at the 4th percentile of METS-VF were significantly higher than those at the 1st percentile (*P* < 0.05). In contrast, we observed significantly lower OC values at the 4th percentile of METS-VF (*P* < 0.05). Trend analyses also confirmed the results of increased BMC levels and decreased OC levels in METS-VF quartiles (*P <* 0.05, Figure 2).

### 3.3 Logistic regression analysis of METS-VF with BMC

After accounting for age, sex, smoking, alcohol consumption, and physical activity (Model 3), METS-VF was significantly associated with a lower risk of low BMC when treated as a continuous variable (OR = 0.48, 95% CI: 0.32–0.74, *P* = 0.001; Supplementary Table S2). When METS-VF was treated as a categorical variable, higher METS-VF quartiles were associated with a reduced risk of low BMC. Specifically, compared with the Q1 group, the risk of low BMC in the Q4 group was significantly lower, with an OR of 0.17 (95% CI: 0.05–0.49, *P* = 0.004; Table 2). In sex-stratified analyses, this association was significant in both boys and girls, although the estimates should be interpreted cautiously because of sparse events (OR_boys_ = 0.03, 95% CI: 0.01–0.46, *P* = 0.007; OR_girls_ = 0.20, 95% CI: 0.06–0.64, *P* = 0.005).

**Table 2:** Firth logistic regression of the relationship between METS-VF and BMC in adolescents.

Variables	Model 1		Model 2		Model 3	
	OR (95% CI)	*P*-values	OR (95% CI)	*P*-values	OR (95% CI)	*P*-values
Q1(total)	Ref.		Ref.		Ref.	
Q2	1.14 (0.78, 1.67)	0.507	0.83 (0.53, 1.29)	0.404	0.93 (0.44, 1.93)	0.846
Q3	0.63 (0.41, 0.96)	**0.031**	0.43 (0.27, 0.70)	**<0.001**	0.32 (0.14, 0.71)	**0.005**
Q4	0.16 (0.08, 0.29)	**<0.001**	0.12 (0.06, 0.23)	**<0.001**	0.17 (0.05, 0.49)	**<0.001**
Q1(boys)	Ref.		Ref.		Ref.	
Q2	1.40 (0.72, 2.73)	0.322	1.70 (0.79, 3.71)	0.173	1.19 (0.31, 4.70)	0.795
Q3	0.89 (0.40, 1.88)	0.764	0.74 (0.29, 1.76)	0.501	0.24 (0.04, 1.13)	0.072
Q4	0.14 (0.03, 0.46)	**<0.001**	0.13 (0.02, 0.47)	**<0.001**	0.03 (0.01, 0.46)	**0.007**
Q1(girls)	Ref.		Ref.		Ref.	
Q2	0.56 (0.33, 0.96)	**0.034**	0.53 (0.30, 0.93)	**0.026**	0.89 (0.36, 2.20)	0.803
Q3	0.26 (0.14, 0.45)	**<0.001**	0.23 (0.12, 0.42)	**<0.001**	0.30 (0.11, 0.77)	**0.012**
Q4	0.09 (0.04, 0.19)	**<0.001**	0.08 (0.03, 0.17)	**<0.001**	0.20 (0.06, 0.64)	**0.005**

Model 1 adjusted for none. Model 2 adjusted for age and sex; Model 3 adjusted for age, sex, smoking, alcohol consumption and physical activity.

OR, odds ratio; CI, confidence interval. Bold values indicate statistical significance (P < 0.05).

### 3.4 Associations of METS-VF with OC and CTX

**Figure 3:**
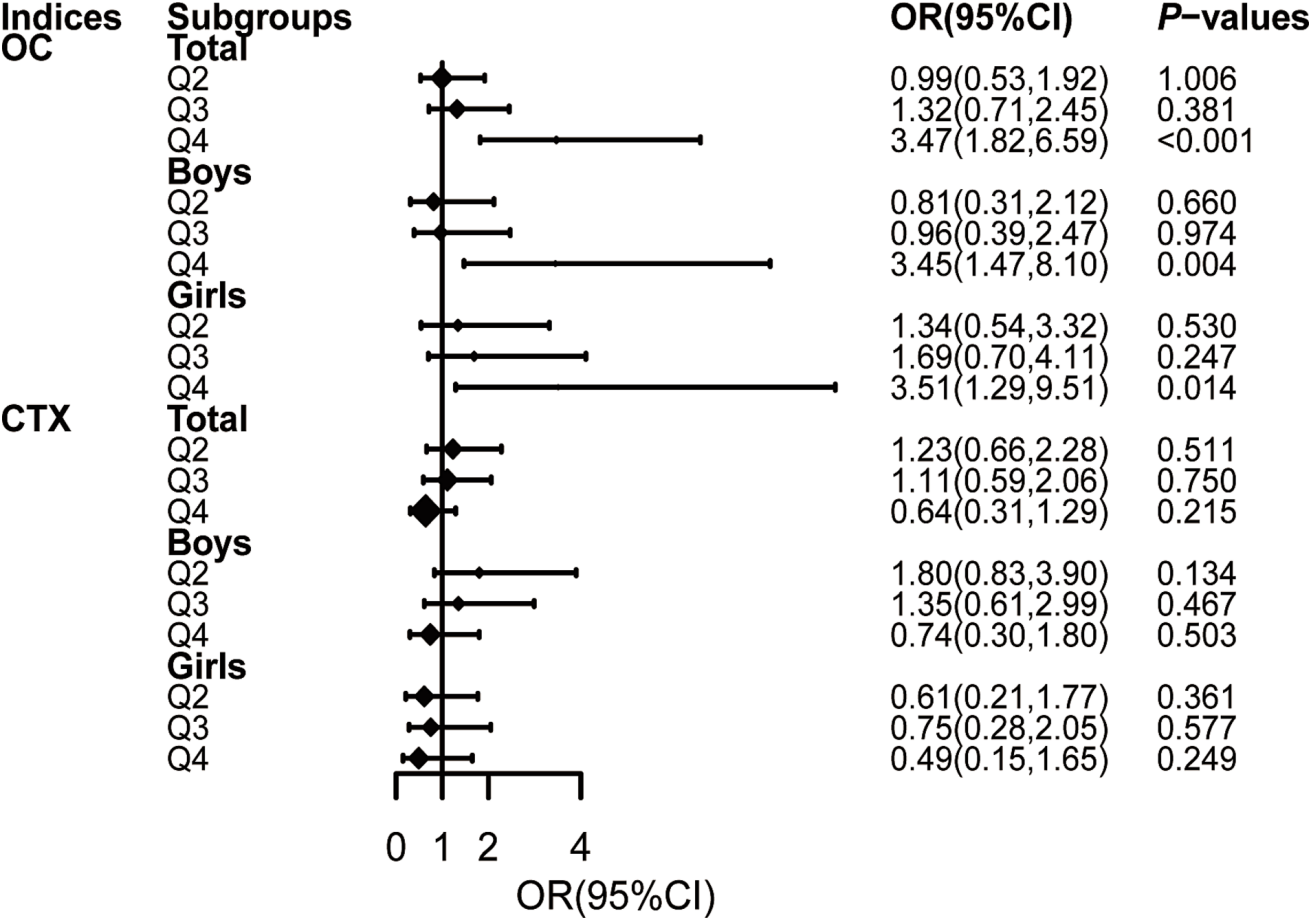
Dose–response relationships between METS-VF and BMC (A), OC (B), and CTX (C) in adolescents. METS-VF, Metabolic Score for Visceral Fat; BMC, bone mineral content; OC, osteocalcin; CTX, C-terminal telopeptide of type I collagen.

Using the 25th percentile as the cut-point, participants were classified into low and high groups for OC and CTX before logistic regression analysis. After adjusting for age, sex, smoking, alcohol consumption and physical activity (Model 3), compared with the Q1 group, the risk of low OC in the Q4 group was higher (OR = 3.47, 95% CI: 1.82–6.59, *P* < 0.001; Figure 3). No significant association was observed between METS-VF and CTX (*P* > 0.05).

### 3.5 Non-linear dose–response relationships

**Figure 4:**
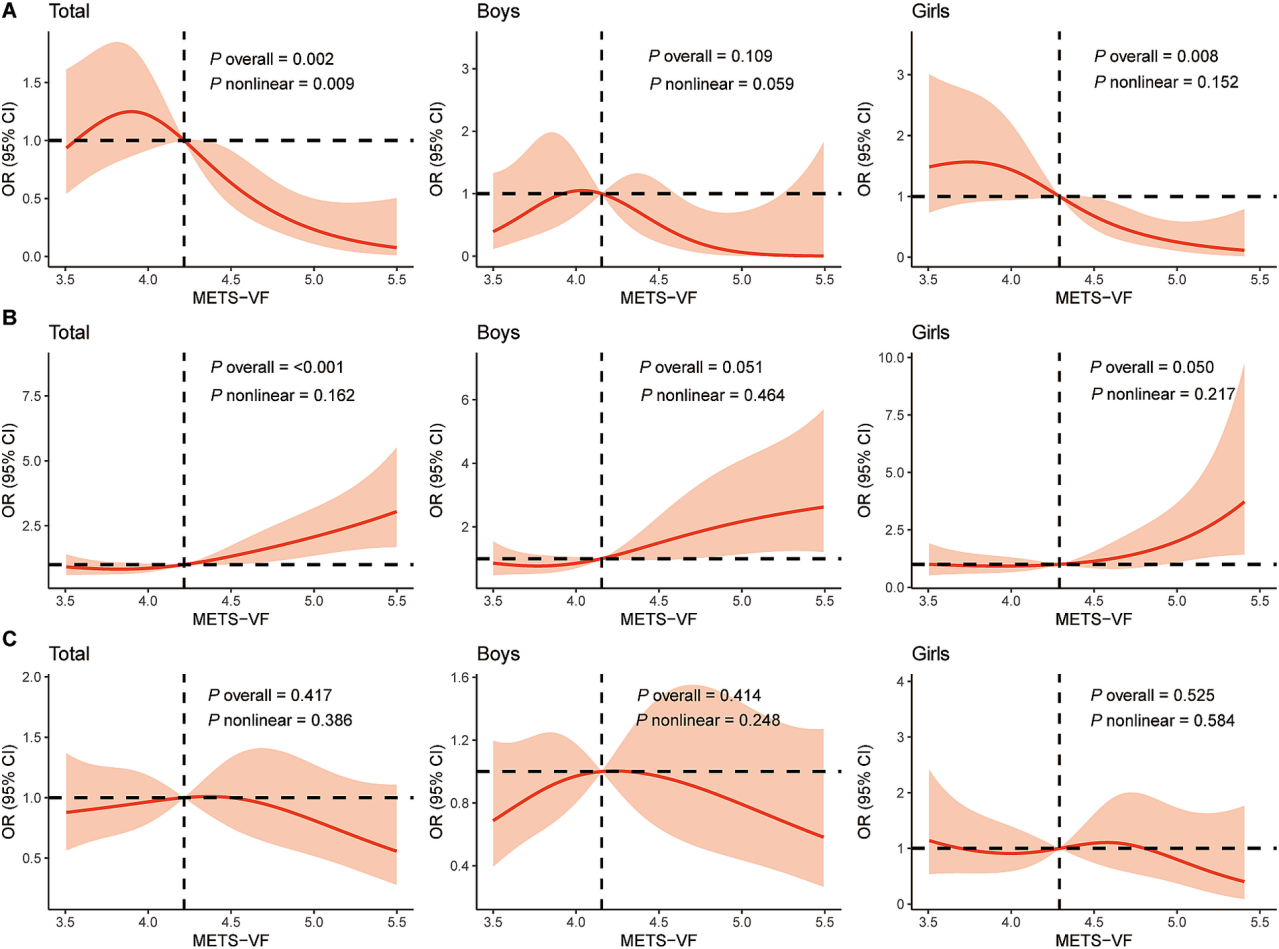
Dose–response relationships between METS-VF and BMC (G), OC (H), and CTX (I) in adolescents. METS-VF, Metabolic Score for Visceral Fat; BMC, bone mineral content; OC, osteocalcin; CTX, C-terminal telopeptide of type I collagen.

RCS curves were drawn to research the trends between METS-VF and BMC, OC, and CTX. After adjusting all confounding factors in Model 3, the results indicated that there was an inverted U-shaped non-linear relationship between METS-VF and BMC (*P-*overall = 0.002, *P-*nonlinear = 0.009; Figure 4A). However, sex-stratified analyses revealed a significant linear association in girls (*P*-nonlinear = 0.152; *P*-overall = 0.008) but no significant association in boys. Furthermore, a significant linear relationship was observed between METS-VF and OC in the overall population (*P*-nonlinear = 0.162; *P*-overall < 0.001), whereas no significant linear association was found for CTX (*P*-nonlinear = 0.386; *P*-overall = 0.417). (Figures 4B,C).

### 3.6 Receiver operating characteristic analysis

**Figure 5:**
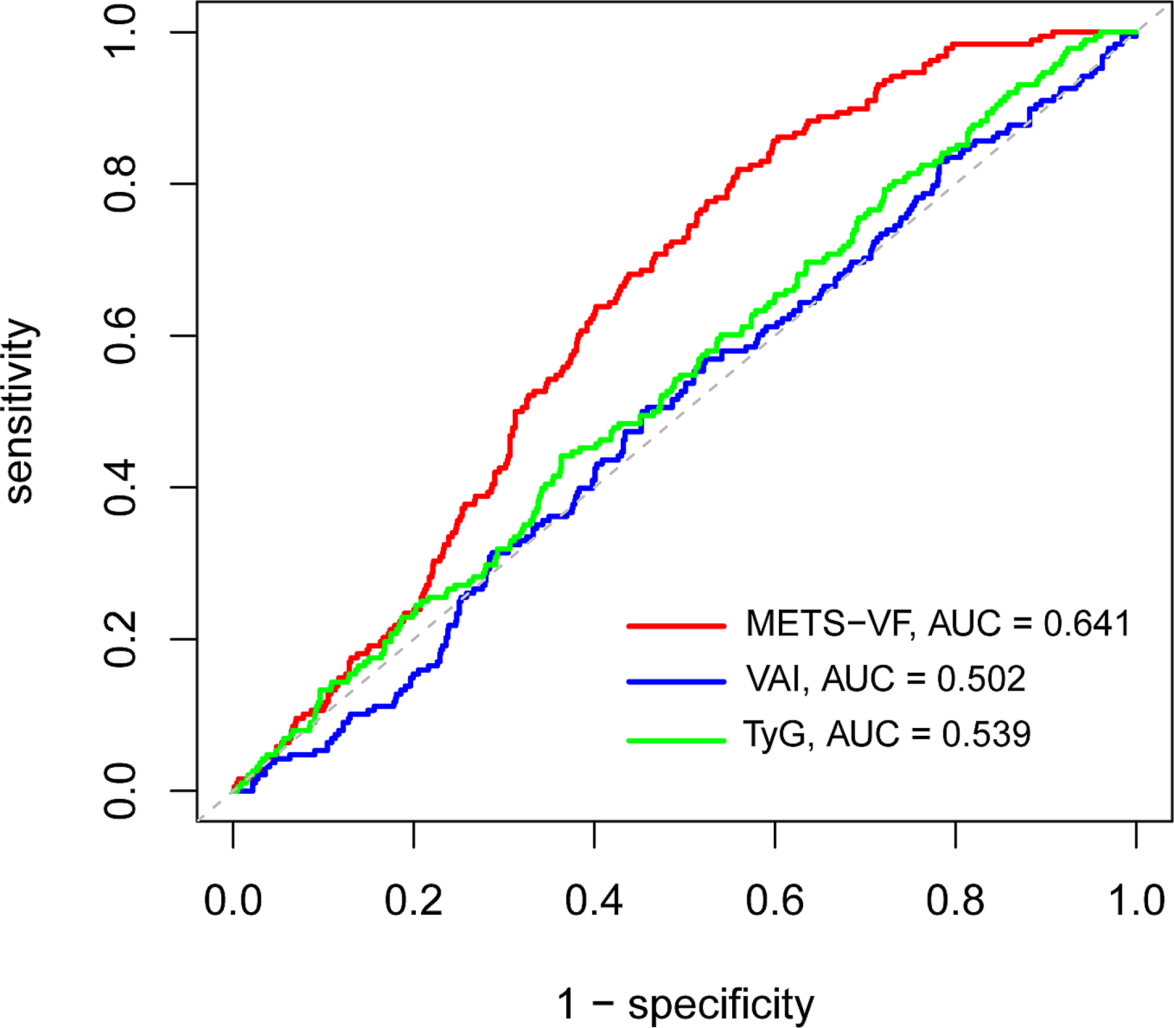
Receiver operating characteristic curves of low bone mass by different indices. The predictive performance of different indices for low bone mass was shown based on the receiver operating characteristic curves. METS-VF, Metabolic Score for Visceral Fat; VAI, visceral adiposity index; TyG^*^, triglyceride-glucose index.

The ROC curve and the AUC value demonstrated that METS-VF exhibited the better diagnostic capability for low BMC, surpassing the visceral adiposity index (VAI), and triglyceride-glucose index (TyG). (Figure 5 and Table 3). METS-VF showed the largest AUC among the three indices and demonstrated statistically significant discriminatory ability for low BMC (*P* < 0.001). The AUC (95% CI), sensitivity, specificity, Youden index, and optimal cut-off criterion of METS-VF were 0.641 (0.605, 0.678), 0.819, 0.441, 0.260, and METS-VF ≤ 4.510, respectively. The AUC of METS-VF was significantly higher than those of VAI and TyG according to DeLong’s test (both *P* < 0.001). For low OC and low CTX, Supplementary Tables S1, S2 present the predictive performance of the three obesity-related indices, with corresponding ROC curves shown in Supplementary Figures S1, S2. The AUCs of METS-VF for predicting low OC and low CTX were 0.559 (95% CI: 0.522–0.596) and 0.541 (95% CI: 0.506–0.575), respectively (all *P* < 0.05).

**Table 3:** The predictive performance of different obesity-related indices for low BMC in adolescents.

Indices	C-statistic (95%CI)	Sensitivity	Specificity	Youden index	Cut-off	*P*-values	*P* for comparison
METS-VF	0.641(0.605,0.678)	0.819	0.441	0.260	4.510	**<0.001**	Ref.
VAI	0.502 (0.459,0.546)	0.830	0.218	0.047	0.672	<0.919	**<0.001**
TyG	0.539(0.495,0.582)	0.441	0.637	0.078	8.058	<0.082	**<0.001**

CI, confidence interval; BMC, bone mineral content; METS-VF, Metabolic score for visceral fat; VAI, visceral adiposity index; TyG^*^, triglyceride-glucose index. Bold values indicate statistical significance (P < 0.05).

### 3.7 Stratified analysis

In analyses stratified by BMI-based weight status, METS-VF was not significantly associated with low BMC, low OC, or low CTX in either the normal-weight or overweight/obesity group. The interaction terms were also not statistically significant (Supplementary Figure S3).

## 4 Discussion

In this cross-sectional study of Chinese adolescents, higher METS-VF was associated with a lower risk of low BMC, but it was also associated with a higher risk of low OC. No significant association was observed between METS-VF and CTX. RCS analyses showed a nonlinear association between METS-VF and low BMC in the total population and a linear association with low OC, whereas no clear association was found for low CTX. BMI-based stratified analyses did not show significant subgroup associations or interactions. Overall, these findings suggest that METS-VF is related to adolescent bone health, with different patterns for bone mass and bone metabolism markers.

The association between METS-VF and BMC may reflect the combined effects of body size, mechanical loading, and visceral adiposity. The present study found that higher METS-VF was associated with higher BMC. This is partly in line with the Framingham Osteoporosis Study (19), which reported positive associations between VAT and bone parameters; however, those associations were attenuated after adjustment for BMI or body weight. Therefore, the observed association should be interpreted in the context of overall body size and skeletal mechanical loading, rather than as evidence of an independent effect of visceral adiposity alone. Similarly, the Amirkola Health and Aging Study (20) found a positive association between VAT and BMD. Moreover, our study revealed a nonlinear, inverted U-shaped relationship between METS-VF and BMC. A non-linear association between visceral mass index and lumbar BMD was found in a study of younger adults which is consistent with the present findings (21). Similar evidence has been documented in observational and Mendelian randomization studies examining the association between VAT and overall fracture risk (22). Excessive obesity leads to a reduction in bone mass. Andrea Palermo et al. found that the protective effect of BMI on fracture is attenuated within a specific range, suggesting that the accumulation of visceral fat may have an important influence on the association between BMI and fracture risk (23). Sex-stratification revealed a positive correlation between METS-VF and BMC only in girls, suggesting that sex differences may play a vital role in the relationship between adiposity and bone. A study examining the relationship between lean body mass and fat mass and BMD displayed that for boys, lean body mass was a key factor influencing whole-body BMD; for girls, the effect of fat mass was more pronounced (24). Sex-stratified RCS analyses showed broadly similar patterns, and the same result of linear correlation between visceral adiposity and bone parameters after sex stratification was shown in a study of the relationship between A Body Shape Index and bone mineral density in adolescents (25).

The associations of METS-VF with OC and CTX suggest that visceral adiposity may be related to bone turnover, particularly bone formation. Bone metabolism markers include markers of bone formation and resorption, which are also collectively referred to as markers of bone turnover. Bone metabolism increases during adolescence, so understanding the status of serum bone metabolism markers in adolescents is beneficial for determining the level of bone tissue development. OC is a marker of bone formation, whereas CTX reflects osteoclast-mediated bone resorption. In this study, higher METS-VF was associated with lower OC levels and a higher risk of low OC, while no significant association was observed between METS-VF and CTX after adjustment for covariates. These findings suggest that METS-VF may be more closely related to bone formation than bone resorption in adolescents. This may be partly explained by the effects of increased adiposity on bone marrow mesenchymal stem cell differentiation. Previous studies have shown that obesity may increase bone marrow adiposity and promote adipocyte differentiation, while suppressing osteoblast differentiation (26). Changes in the arrangement of adipose tissue with aging, particularly an increase in visceral fat, contribute to a decrease in BMD (27). For CTX, the null association observed in our study is consistent with previous evidence showing that the association between BMI and CTX became non significant after adjustment for covariates (28). Therefore, the relationship between METS-VF and CTX in adolescents remains uncertain and may require further investigation in larger studies.

The coexistence of higher BMC and lower OC among adolescents with higher METS-VF may appear contradictory, but it is biologically plausible given the complex relationship between VAT and bone health. During adolescence, modest increases in fat mass and body weight may enhance skeletal loading, which promotes bone mineral accrual and may partially explain the higher BMC observed at higher METS-VF levels. In contrast, visceral adiposity may exert unfavorable metabolic effects on bone formation. Excess visceral fat secretes pro-inflammatory cytokines, such as IL-1, IL-6, and TNF-*α*, which may adversely affect bone through inflammatory pathways. It also secretes adipokines, such as leptin and adiponectin, which are involved in bone metabolism by regulating osteoblast and osteoclast activity. Previous studies have reported both positive and negative associations between leptin and BMD (29, 30). Lipocalin stimulates RANKL through MAPK signaling and inhibits the expression of OPG in human osteoblasts, which indirectly leads to bone loss (31). In addition, insulin resistance may also affect bone metabolism through visceral adiposity (32). Emerging evidence also suggests that broader metabolic and environmental factors may have systemic implications for health outcomes and may indirectly influence skeletal health (33, 34). Therefore, adolescent bone health should be interpreted within the broader context of metabolic homeostasis and environmental exposure. The METS-VF, a measure of abdominal fat distribution, is commonly used to study the relationship between centripetal obesity and bone health. Hence the effects of adiposity and muscle mass on bone health may be even more important. In light of this, improved dietary habits and physical activity levels in adolescents are indispensable, not only to benefit bone development, but also to improve overall bone health.

The strength of this study is that it is the first to examine the relationship between METS-VF and bone health in adolescents. However, a major limitation of this study is that pubertal stage, sex hormone levels, dietary calcium and vitamin D intake, and sun exposure were not collected. Future studies should include these factors to further clarify the relationship between visceral adiposity and adolescent bone health. Secondly, BMC was assessed using BIA rather than DXA, the gold standard for BMC measurement; therefore, the findings should be interpreted cautiously and validated in future studies using DXA. Thirdly, this was a cross-sectional study conducted among adolescents aged 12–18 years, which does not allow causal relationships between METS-VF and bone metabolism to be established. Further prospective cohort studies are needed to validate these findings. Finally, because the sample size was determined by the availability of complete data from multiple survey waves, no *a priori* sample size calculation was performed. Thus, some subgroup analyses may have been underpowered and should be interpreted cautiously.

In conclusion, our findings demonstrate that METS-VF is associated with adolescent bone health, showing an inverted U-shaped nonlinear relationship with low BMC and a positive association with low OC, but no significant association with low CTX. Sex-specific differences in these associations further highlight the complexity of the link between visceral adiposity and adolescent bone metabolism. As a valid indicator of visceral fat, METS-VF provides a useful tool for exploring adiposity-bone interactions in adolescent populations.

## Data Availability

The datasets generated and analyzed during the current study are not publicly available due to ethical and privacy restrictions, but are available from the corresponding author on reasonable request.
