# Supplementary material for: Impact of metabolic score for visceral fat on bone health in adolescent population: a cross-sectional study perspective
Source: Front Nutr. 2026 Jun 30;13:1822235. doi: 10.3389/fnut.2026.1822235 (PMC13365040; doi:10.3389/fnut.2026.1822235)
Supplement: Supplementary file 1 [file Supplementary_file_1.DOCX]

Supplementary Material

# Supplementary Figures and Tables

Table S1 General characteristics of participants included in and excluded from the final analytic sample.

| Characteristics | Included | Excluded | *P*-value |  |
| --- | --- | --- | --- | --- |
| N | 1206 | 1492 |  |  |
| Age(years） | 14.70±1.27 | 15.37±1.59 | **<0.001** |  |
| Sex, n (%) |  |  | **<0.001** |  |
| male | 680(56.4) | 956(64.1) |  |  |
| female | 526(43.6) | 536(35.9) |  |  |
| BMI(Kg/m2) | 20.60±3.94 | 20.85±3.95 | 0.103 |  |
| WC (cm) | 75.15±10.66 | 75.09±10.68 | 0.892 |  |
| SBP (mmHg) | 113.09±11.74 | 111.98±11.79 | **0.021** |  |
| DBP (mmHg) | 70.02±7.90 | 68.37±8.28 | **<0.001** |  |
| Smoking n (%) |  |  | **<0.001** |  |
| Yes | 206 (17.9) | 323 (26.8) |  |  |
| No | 947 (82.1) | 884 (73.2) |  |  |
| Alcohol consumption n (%) |  |  | 0.455 |  |
| Yes | 367 (31.9) | 401 (33.5) |  |  |
| No | 782 (68.1) | 797 (66.5) |  |  |
| FPG (mg/dL) | 90.45±10.69 | 91.44±21.77 | 0.169 |  |
| TG^a^ (mg/dL) | 79.71 [62.88, 105.40] | 83.26 [65.54, 107.17] | 0.007 |  |
| TC (mg/dL) | 146.45±28.43 | 161.68±40.28 | **<0.001** |  |
| HDL (mg/dL) | 52.58±10.21 | 52.92±16.78 | 0.529 |  |
| LDL (mg/dL) | 73.24±24.03 | 90.30±30.71 | **<0.001** |  |
| BMC(Kg) | 2.63±0.51 | 2.67±0.51 | 0.051 |  |
| OC (ng/mL) | 20.14±13.87 | 20.15±14.06 | 0.980 |  |
| CTX (pg/mL) | 2344.93±1751.31 | 3071.17±1688.42 | **<0.001** |  |
| Abbreviations: BMI: body mass index; WC: waist circumference; SBP: Systolic Blood Pressure; DBP: Diastolic Blood Pressure; FPG: Fasting Plasma Glucose; TC: Total Cholesterol; TG*: Triglyceride; HDL-C: High-density lipoprotein cholesterol; LDL-C: Low-density lipoprotein cholesterol: bone mineral content; OC: osteocalcin; CTX: C-terminal telopeptide of type I collagen. Data are presented as means ± SD or median (interquartile range) for continuous variables and numbers (%) for categorical variables;  *P*-values are calculated by ANOVA and Kruskal–Wallis tests for continuous variables and Chi-square tests for categorical variables.  ᵃThe P-value for TG was calculated using the Kruskal–Wallis test. | | | | |

Table S2 Logistic regression of the relationship between METS-VF and BMC in adolescents.

| Variables | Model 1 | | | Model 2 | | | Model 3 | | |
| --- | --- | --- | --- | --- | --- | --- | --- | --- | --- |
|  | N_low BMC_/N_Q_ | OR (95%CI) | *P*-values | N_low BMC_/N_Q_ | OR (95%CI) | *P*-values | N_low BMC_/N_Q_ | OR (95%CI) | *P*-values |
| Continuous | 188/1206 | 0.47(0.38,0.59) | **<0.001** | 188/1206 | 0.42(0.32,0.54) | **<0.001** | 73/407 | 0.48(0.32,0.74) | 0.001 |
| Quartiles |  |  |  |  |  |  |  |  |  |
| Q1(total) | 63/301 | Ref. |  | 63/301 | Ref. |  | 26/118 | Ref. |  |
| Q2 | 70/302 | 1.14(0.78,1.68) | 0.506 | 70/302 | 0.91(0.60,1.39) | 0.665 | 29/101 | 1.18(0.59,2.35) | 0.945 |
| Q3 | 43/302 | 0.63(0.41,0.96) | 0.032 | 43/302 | 0.48(0.30,0.77) | **0.002** | 14/104 | 0.42(0.19,0.91) | **0.027** |
| Q4 | 12/301 | 0.16(0.08,0.30) | **<0.001** | 12/301 | 0.14(0.07,0.27) | **<0.001** | 4/84 | 0.19(0.06,0.58) | **0.004** |
| Q1(boys) | 19/211 | Ref. |  | 19/211 | Ref. |  | 9/77 | Ref. |  |
| Q2 | 19/156 | 1.40(0.72,2.75) | 0.325 | 19/156 | 2.01(0.96,4.23) | 0.066 | 8/52 | 2.62(0.70,9.80) | 0.153 |
| Q3 | 11/138 | 0.88(0.40,1.90) | 0.736 | 11/138 | 0.96(0.41,2.23) | 0.924 | 5/50 | 0.64(0.16,2.60) | 0.528 |
| Q4 | 2/175 | 0.12(0.03,0.51) | **0.004** | 2/175 | 0.13(0.03,0.59) | **0.008** | 0/45 |  |  |
| Q1(girls) | 44/90 | Ref. |  | 44/90 | Ref. |  | 17/41 | Ref. |  |
| Q2 | 51/146 | 0.56(0.33,0.96) | **0.034** | 51/146 | 0.55(0.32,0.95) | **0.031** | 21/49 | 0.90(0.37,2.18) | 0.818 |
| Q3 | 32/164 | 0.25(0.14,0.45) | **<0.001** | 32/164 | 0.24(0.14,0.44) | **<0.001** | 9/54 | 0.29(0.11,0.76) | **0.012** |
| Q4 | 10/126 | 0.09(0.04,0.19) | **<0.001** | 10/126 | 0.09(0.04,0.19) | **<0.001** | 4/39 | 0.20(0.06,0.69) | **0.011** |
| Note: Model 1 adjusted for none. Model 2 adjusted for age and sex; Model 3 adjusted for age, sex, smoking, alcohol consumption and physical activity.  Abbreviations: OR: odds ratio; CI: confidence interval  N_low BMC_, number of participants with low BMC; N_Q_, number of participants included in the model. | | | | | | | | | |

Table S3 The predictive performance of different obesity-related indices for low OC in adolescents

| Indices | C-statistic(95%CI) | Sensitivity | Specificity | Youden index | Cut-off | *P*-values | *P* for comparison |
| --- | --- | --- | --- | --- | --- | --- | --- |
| METS-VF | 0.559(0.522,0.596) | 0.420 | 0.708 | 0.128 | 4.708 | **0.002** | Ref. |
| VAI | 0.574 (0.538,0.609) | 0.619 | 0.510 | 0.129 | 0.951 | **<0.001** | 0.495 |
| TyG | 0.522(0.486,0.557) | 0.869 | 0.195 | 0.064 | 8.601 | 0.229 | 0.202 |
| *P* values in bold are<0.05.  Abbreviations: CI: confidence interval; METS-VF: Metabolic Score for Visceral Fat; VAI: visceral adiposity index; TyG*: triglyceride-glucose index. | | | | | | | |

| 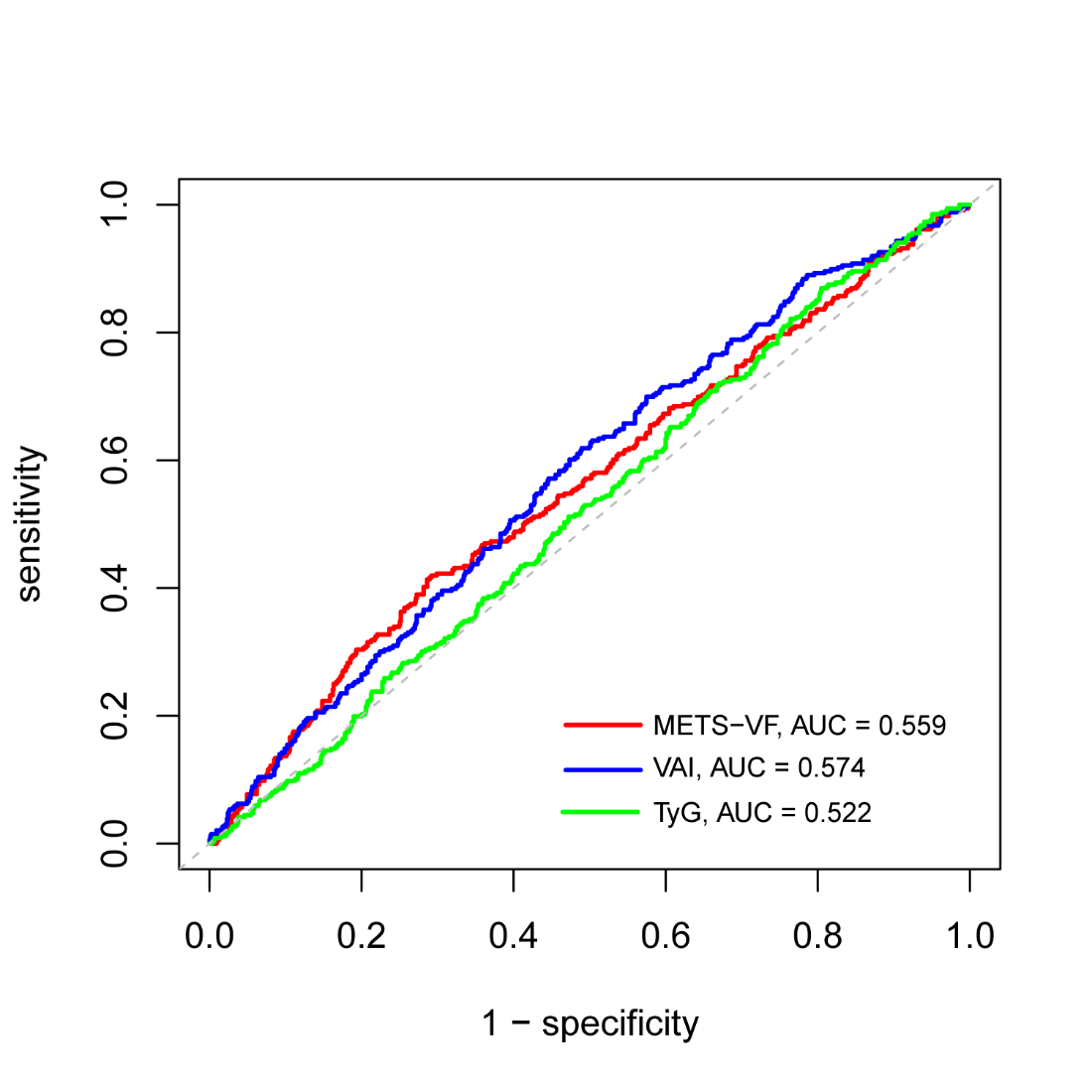 |
| --- |
| Figure S1. Receiver operating characteristic curves of low OC by different indices. The predictive performance of different indices for low bone mass was shown based on the receiver operating characteristic curves. |

Abbreviations: OC: osteocalcin; METS-VF: Metabolic Score for Visceral Fat; VAI: visceral adiposity index; TyG^*^: triglyceride-glucose index.

Table S4 The predictive performance of different obesity-related indices for low CTX in adolescents

| Indices | C-statistic(95%CI) | Sensitivity | Specificity | Youden index | Cut-off | *P*-values | *P* for comparison |
| --- | --- | --- | --- | --- | --- | --- | --- |
| METS-VF | 0.541(0.506,0.575) | 0.675 | 0.412 | 0.087 | 4.544 | **0.022** | Ref. |
| VAI | 0.568 (0.533,0.603) | 0.402 | 0.709 | 0.112 | 0.785 | **<0.001** | 0.182 |
| TyG | 0.537(0.502,0.573) | 0.683 | 0.399 | 0.082 | 8.056 | **0.040** | 0.908 |
| *P* values in bold are<0.05.  Abbreviations: CI: confidence interval; METS-VF: Metabolic Score for Visceral Fat; VAI: visceral adiposity index; TyG*: triglyceride-glucose index. | | | | | | | |

| 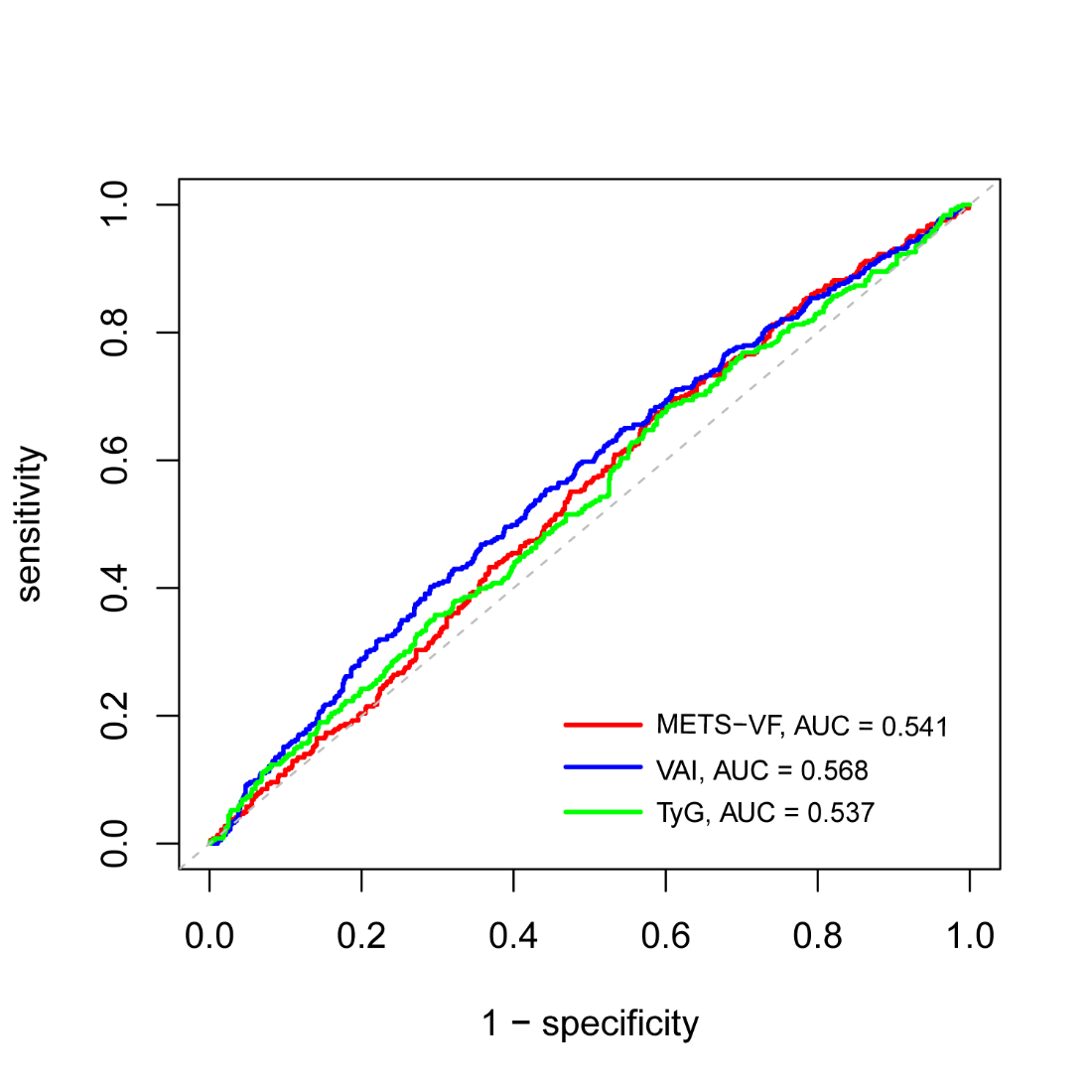 |
| --- |
| Figure S2. Receiver operating characteristic curves of low CTX by different indices. The predictive performance of different indices for low bone mass was shown based on the receiver operating characteristic curves. |

Abbreviations: CTX: type I collagen carboxy-terminal peptide. METS-VF: Metabolic Score for Visceral Fat; VAI: visceral adiposity index; TyG^*^: triglyceride-glucose index.


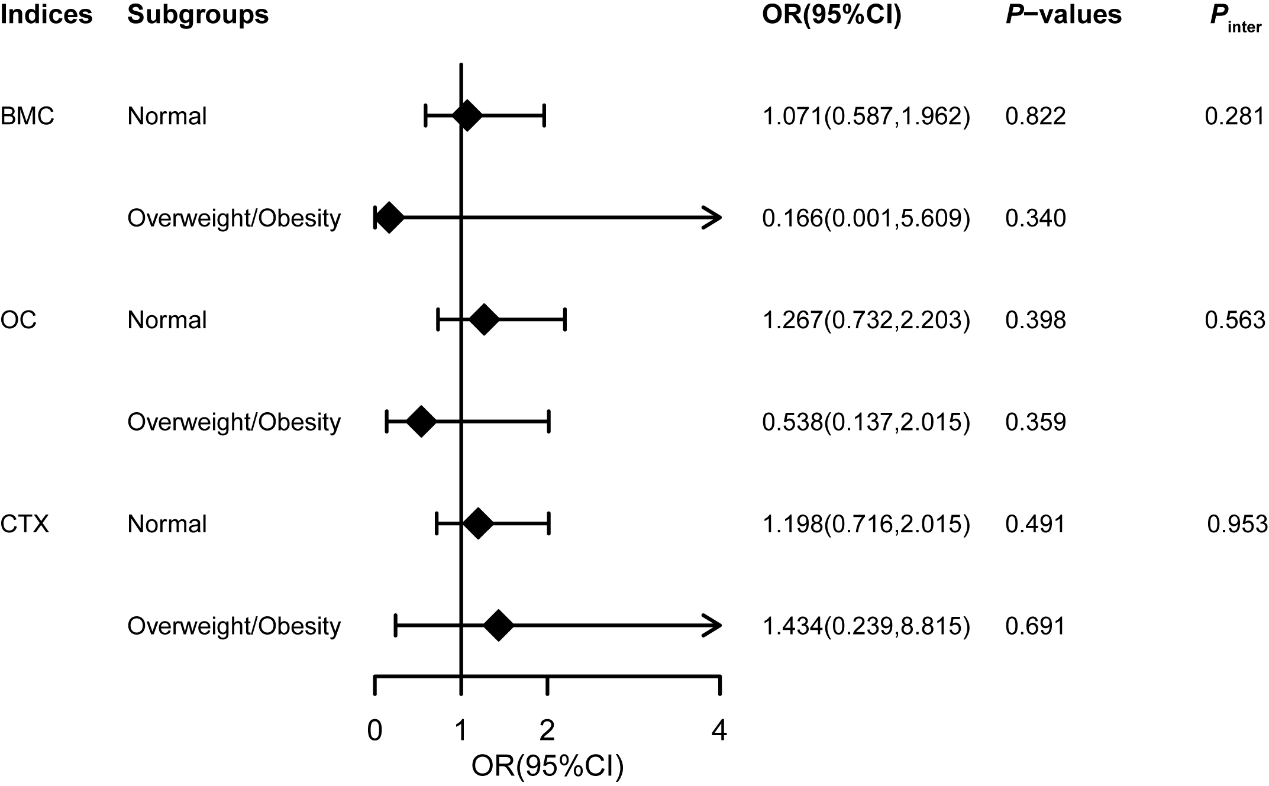


Figure S3 Stratified associations of METS-VF with low OC, low BMC, and low CTX according to weight status.

Odds ratios were estimated using Firth logistic regression adjusted for age, sex, smoking, alcohol consumption, and physical activity.
